# How to Assess the Headache—Sleep Disorders Comorbidity in Children and Adolescents

**DOI:** 10.3390/jcm10245887

**Published:** 2021-12-15

**Authors:** Agnese Onofri, Michela Ada Noris Ferilli, Elisabetta Tozzi, Fabiana Ursitti, Giorgia Sforza, Luca Olivieri, Martina Proietti Checchi, Federico Vigevano, Massimiliano Valeriani, Romina Moavero

**Affiliations:** 1 Neuropsychiatric Clinic—Child Headache Center, Department of Life, Health and Environmental Sciences, San Salvatore Hospital L’Aquila, University of L’Aquila, 67100 L’Aquila, Italy; agnese.onofri@graduate.univaq.it (A.O.); elisabetta.tozzi@univaq.it (E.T.); lucaolivieri.lu@outlook.it (L.O.); 2 Neuroscience Department, Bambino Gesù Children’s Hospital IRCCS, Piazza di Sant’Onofrio 4, 00165 Rome, Italy; fabiana.ursitti@opbg.net (F.U.); giorgia.sforza@opbg.net (G.S.); federico.vigevano@opbg.net (F.V.); massimiliano.valeriani@opbg.net (M.V.); romina.moavero@opbg.net (R.M.); 3 Unit of Clinical Psychology, Neuroscience Department, Bambino Gesù Children’s Hospital IRCCS, 00165 Rome, Italy; martina.proietti@opbg.net; 4 Denmark Neurology Unit, Center for Sensory-Motor Interaction, Aalborg University, 9220 Aalborg, Denmark; 5 Child Neurology Unit, Systems Medicine Department, Tor Vergata University Hospital of Rome, 00133 Rome, Italy

**Keywords:** sleep disorders, primary headaches, migraine, tension-type headache, comorbid conditions, children

## Abstract

Sleep disorders and primary headaches are frequent health problems in childhood, and they are often comorbid in an individual, linked by a mutual and complex relationship. This comorbidity is frequent and well-documented, but the available literature is usually biased in favor of one aspect or another, mainly depending on the expertise of the authors. The aim of this paper is to review existing literature on the diagnostic assessment of comorbid primary headaches and sleep disorders, so as to propose practical suggestions to accurately investigate the presence of comorbid conditions in children evaluated for primary headaches or for sleep disorders.

## 1. Introduction

Sleep disorders and headaches are frequent health problems in childhood, with primary headaches occurring in 12% of the pediatric population [1] and with 24% of children experiencing at least one type of sleep problem [2]. Sleep disturbances and headaches are often comorbid in an individual, and they are linked by a mutual and complex relationship. Headaches might be a consequence of disrupted sleep, with sleep alterations acting as trigger factors for attacks, especially in subjects who suffer migraines. Sleep disorders might also increase the severity of attacks and favor chronicization [3]. Furthermore, the circadian phenotype and the quality of sleep can influence migraine characteristics [4,5]. Inversely, headaches can also cause sleep problems, especially when nocturnal attacks interrupt sleep [6]. The high comorbidity rate between sleep disorders and headaches might indicate different underlying reasons. First, the alteration of the serotoninergic system, involved both in nociception and sleep regulation, might play a role [7]. Furthermore, both disorders could represent an expression of a common pathogenic process, with common cerebral structures and signaling pathways (including the hypothalamus, raphe nuclei, and serotoninergic system) involved in both sleep and migraines [6,8,9,10]. This complex correlation between headache and sleep disturbances related to common neurophysiological processes has important implications for the treatment of both conditions. In fact, drugs used in the treatment of primary headache can influence the physiology of sleep, as well as the treatment of sleep disorders, either with behavioral or pharmacological approach, can significantly improve migraine [11,12]. Last, common risk factors such as mood and anxiety disorders, related to both headaches and sleep disturbances, might increase the risk of this comorbidity [13]. Due to this complex and mutual relationship, a correct assessment of both disorders is necessary for planning an individualized treatment strategy addressing sleep disorders and headaches at the same time, thus ameliorating the quality of life of patients. Although this comorbidity is frequent and well-documented, the available literature is usually biased in favor of one aspect or another, mainly depending on the expertise of the authors. Several studies rigorously assessed migraines by following the diagnostic criteria of the International Classification of Headache Disorders (ICHD), but sleep disorders are poorly documented. Conversely, there are some studies focused on sleep disorders that use standardized sleep questionnaires or neurophysiologic studies, but the diagnosis of migraine diagnosis is not accurate [14,15,16].

The aim of this paper is to review existing literature on the diagnostic assessment of comorbid primary headaches and sleep disorders, so as to make practical suggestions for accurately investigating the presence of comorbid conditions in children evaluated for primary headaches or for sleep disorders.

## 2. Materials and Methods

The results of this review are reported according to the preferred reporting items for reviews and meta-analyses (PRISMA) and adhere to a structured review protocol [17]. To establish the research question, the PICO-model PICOS [18] (Patient, Intervention, Comparison, Outcome), according to the PRISMA guidelines was used: Patient: children with sleep disorders and primary headache, Intervention: review existing literature on the diagnostic assessment of comorbid primary headache and sleep disorders, Comparison: n.a., Outcome: propose some practical suggestion to accurately investigate the presence of comorbid conditions in children evaluated for primary headache or for sleep disorders.

### Search Strategy and Article Selection

Two authors (A.O. and E.T.) performed a comprehensive search of four databases, PubMed, Embase, Cochrane, and Web of Science, using the following search strategy: “primary headache” OR “migraine” OR, AND “sleep” OR “sleep disorders” OR “sleep disturbance” OR, AND “diagnosis”.

Studies were initially included if they:

(1) Involved individuals with headaches and sleep disorders;
(2) Involved children and adolescents up to 18 years of age;
(3) Reported the diagnostic evaluation methods for headaches and sleep disorders;
(4) Were written in English;
(5) Were published within 10 years of the search date (January 2010–December 2020), which was considered a sufficient period to capture publications with the most reliable, appropriate, and up-to-date diagnostic procedures.

We excluded:

(1) Review articles, case reports, letters, metanalysis, and books
(2) Treatment studies (both pharmacological and non-pharmacological);
(3) Studies in which subjects presented other neurological disorders, such as epilepsy, or wherein the presence of headaches was addressed chiefly as a symptom in the context of other general medical conditions, or that dealt with neurodevelopmental disorders such as children with intellectual disability, borderline intellectual disability, psychiatric disorders, attention deficit hyperactivity disorder (ADHD), and tics;
(4) Sleep disorders secondary to nocturnal enuresis;
(5) Studies that relied exclusively on neurophysiological methods, since these are recognized and valid methods to study sleep characteristics but represent second-step investigations, as they are not used in the daily clinical practices.

Two authors (A.O. and E.T.) independently screened all the titles and abstracts of studies identified by the initial search. The full text of an article was obtained when either reviewer thought that it might fulfill the inclusion criteria. When there was uncertainty regarding the inclusion of a publication, three additional authors were consulted (M.A.N.F., R.M., and M.V.).

Full articles were reviewed for relevance and articles were excluded if they did not include data relating to the diagnostic methods applied to evaluate the presence of sleep disorders and/or headaches.

## 3. Results

**Figure 1:**
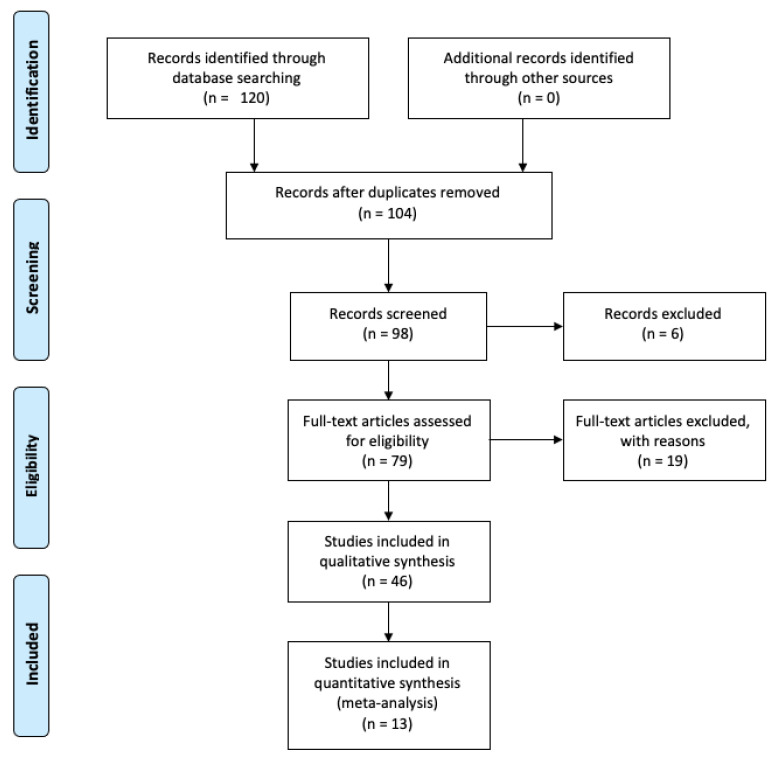
PRISMA flow diagram.

After the initial identification of 120 papers, for the final analysis, we selected 13 manuscripts that fulfilled our inclusion criteria (Figure 1). 

Table 1 shows the main characteristics of all the studies selected for drafting this review.

Overall, these articles reported the results involving 14,674 subjects aged 5–18 years. 

Of the 13 included studies of primary headaches, 11 diagnosed according to ICHD criteria, while the remaining 2 papers used self-diagnosis tools reports [19] or country-specific diagnostic criteria [10]. Included as primary headaches were migraines with (MwA) and without auras (MwoA), chronic migraines (CM), tension-type headaches (TTH), new daily persistent headaches (NDPH), and probable migraines (PM). 

Several papers included the administration of specific tools used to evaluate the severity of headaches (Table 2), such as the Migraine Disabilities Assessment Scale (MIDAS) [19], Pediatric Migraine Disability Assessment Score (PedMIDAS) [20,21,25], and numerical rating scales (NRS) [10,22].

Large variations were noted in the measures and criteria used to define sleep disorders. None of the manuscripts mentioned the diagnostic criteria of the International Classification of Sleep Disorders (ICSD). However, different studies used validated questionnaires, including the Children’s Sleep Habits Questionnaire (CSHQ) [10,26], Sleep Disturbances Scale for Children (SDSC) [24,28], Pediatric Daytime Sleepiness Scale (PDSS) [28], BEARS Sleep Screening Tool [21], and Sleep Hygiene Inventory for Pediatrics (SHIP) [22,23]. In some studies, other questionnaires, including the Patient-Reported Outcomes Measurement Information System (PROMIS) [19], general lifestyle questionnaire [20], and National Comorbidity Survey-Adolescent (NCS-A) [13], were used (Table 3). 

Different types of sleep disorders emerged: insomnia, disturbances of the sleep–wake rhythm, obstructive sleep apnea syndrome, daytime sleepiness, parasomnia, sleep anxiety, hypersomnia, snoring, co-sleeping, and unspecified sleep disorders. 

## 4. Discussion and Conclusions

This review highlights that most researchers applied the ICHD criteria to reach the diagnosis of primary headache, while none of them mentioned the ICSD criteria. As for sleep, only 7 out of 13 analyzed studies used standardized questionnaires, meaning that in almost half of these papers, sleep was investigated through non-validated methods or with general questionnaires not specifically designed for sleep disorders. Furthermore, even when validated, some sleep questionnaires could not be considered as “fully diagnostic”, since they offered only a screening of sleep problems to be studied in more detail. The BEARS is a rapid, yet not diagnostic, screening tool that is easy to use in everyday clinical practice. Similarly, the SHIP questionnaire was specifically developed to highlight clinical concerns about sleep in patients suffering from primary headaches [23]. Overall, we can conclude that most of the selected articles rigorously analyzed only one aspect of the comorbidity, with sleep often representing the “weak” side of the study.

A prompt and correct diagnosis of this comorbidity is of crucial importance. Only treating headaches in a child who is also presenting a sleep disorder cannot produce good results, leading to the classification of the patient as a “non-responder” to first-line treatments. On the other hand, targeting both disorders at the same time can significantly ameliorate the quality of life of patients (and their families) and reduce all symptoms. A recent study by our group (published after the period considered for this review article) aimed at analyzing the relationship between migraines and sleep in a wide sample of pediatric migraine patients [38]. The study was conducted by administering a standardized questionnaire (CSHQ) to patients presenting a definite diagnosis of migraine according to ICHD-3 criteria. The results confirmed that sleep disorders are a very common complaint in pediatric patients with migraines, affecting about 73% of patients. 

Sleep disorders are also included in the “episodic syndromes that may be associated with migraine” and are considered early life expressions of migraine [9,39]. Sleep disorders, such as sleep walking, sleep talking, night terror, and bruxism, are now included in the ICHD-3 classification [40] and considered part of a “migraine syndrome of childhood” [41,42]. Therefore, a correct and precise assessment of symptoms is of crucial importance, and it is worth emphasizing that most sleep disorders are easily assessable through accurate clinical histories and specific questionnaires. In the pediatric age group, it was shown that parental reports are consistent with objective measurements such as actigraphy and polysomnography [43]. 

In conclusion, this review underlines the paucity of data correctly assessing both headache and sleep disorders, thus leaving this comorbidity often underdiagnosed and there-fore undertreated. These results support the need, in the daily clinical practice, for an ex-tensive clinical history and validated assessments, including validated questionnaires and possibly actigraphy and polysomnography, in children searching medical advice for migraine or sleep disorders in order to be able to detect this comorbidity early, thus de-signing the better treatment strategy for the patient.

## Figures and Tables

**Table 1:** Main characteristics of all studies selected for drafting this review.

Ref.	*n*	Age (Years)	Headaches (H)			Sleep Disorders (SD)		Aim of Study
			Type	H Diagnosis	Evaluation of Headache Severity	Type	SD Diagnosis	
Kemper, K.J. et al., 2016 [19]	29	12–18	MwA, MwoA, CM, TTH	Self-report	MIDAS	Sleep disturbances, unspecified	PROMIS sleep disturbances scale	H
Torres-Ferrus, M. et al., 2019 [20]	1619	12–18	PM	ICHD-3 beta version	PedMIDAS	Insomnia Daytime sleepiness	Lifestyle questionnaire	H
Lateef T. et al., 2019 [13]	10,123	13–18	MwA, MwoA	ICHD-3	none	Insomnia	NCS-A interview	H + SD
Cheraghi F. et al., 2018 [10]	198	6–12	M, TTH	Country-specific diagnostic criteria	NRS	Bedtime resistance (BTR) Sleep onset delay (SOD) Sleep duration (SD) Sleep anxiety (SA) Night wakening (NW) Parasomnia (PS) Sleep-disordered breathing (SDB) Daytime sleepiness (DTS)	CSHQ	H + SD
Fonseca E. et al., 2020 [21]	19	10.2 ± 2.9	MwA, MwoA	ICHD-3 beta version	PedMIDAS	Bedtime issues Excessive daytime sleepiness Night awakenings Regularity and duration of sleep Snoring	BEARS sleep screening tool	H
Rabner J. et al., 2017 [22]	527	7–17	M, TTH, NDPH	ICHD-2	NRS	Sleep disturbance, unspecified	SHIP	H + SD
Rabner J. et al., 2017 [23]	1078	7–17	M, TTH, NDPH and mixed headache presentation	ICHD-2	none	Sleep disturbance, unspecified	SHIP	H + SD
Maltese A. et al., 2017 [24]	64	8–12	MwoA, episodic TTH and chronic TTH	ICHD-3	none	Disorders in initiating and maintaining sleep (DIMS) Disorders of arousal (DA) Sleep–wake transition disorders (SWTD)	SDSC	H + SD
Heyer GL. et al., 2014 [25]	52	10–18	M, PM	ICHD-2	PedMIDAS	Sleep disruptions directly related to proximate headache	none	H + SD
Abou-Khadra MK et al., 2013 [26]	18	5–12	M	ICHD-2	none	Bedtime resistance (BTR) Sleep onset delay (SOD) Sleep duration (SD) Sleep anxiety (SA) Night wakening (NW) Parasomnia (PS) Sleep-disordered breathing (SDB) Daytime sleepiness (DTS)	CSHQ	SD
Yilmaz M. et al., 2013 [27]	511	8–15	M, TTH	ICHD-2	none	Poor sleep Nocturnal awakenings Nightmares Apneas	Questionnaire survey	H
Esposito M. et al., 2013 [28]	271	6–13	MwoA	ICHD-2	none	Disorders in initiating and maintaining sleep Sleep breathing disorders Disorders of arousal Sleep–wake transition disorders Disorders of excessive somnolence Nocturnal hyperhidrosis Daytime sleepiness	SDSC, PDSS	H + SD
Carotenuto M. et al., 2011 [29]	181	9.02 ± 0.99	MwoA	ICHD-2	none	Co-sleeping	Clinical interview	H + SD

Abbreviations. Ref: reference; *n*: number of patients; H: headache; SD: sleep disorders; MwA; migraine with aura; MwoA: migraine without aura; CM: chronic migraine; TTH: tension-type headache; PM: probable migraine; M: migraine; NDPH: new daily persistent headache; ICHD: International Classification of Headache Disorders; MIDAS: migraine disabilities assessment scale; PedMIDAS: pediatric migraine disability assessment score; NRS: numerical rating scale; SHIP: sleep hygiene inventory for pediatrics; SDSC: sleep disturbance scale for children; PDSS: pediatric daytime sleepiness scale; PROMIS: patient-reported outcomes measurement information system; NCS-A: National Comorbidity Survey-Adolescent; CSHQ: children’s sleep habits questionnaire; BEARS: B = bedtime issues, E = excessive daytime sleepiness, A = night awakenings, R = regularity and duration of sleep, S = snoring.

**Table 2:** List of tools used to assess migraine severity in the considered papers, with a brief description.

Tool	Description	Structure	Ref.
MIDAS	Instrument for migraine-related disability	The MIDAS score is derived as the sum of missed days from paid work or school, household work, and nonwork activities due to headache. Headache patients answer five questions, scoring the number of days in the past 3 months when activity was limited due to migraine	Stewart W.F. et al., 2001 [30].
PEDMIDAS	Validated questionnaire recognized in the assessment of the disability of childhood and adolescent headaches	The score is a simple composite of the total of six questions related to the impact of headaches on school performance, disability at home, and social/sports functions	Hershey A.D. et al., 2001 [31]
NUMERICAL RATING SCALES	Scale to rate the pain	Eleven-point numerical scale; the child rates the pain from 0 (no pain) to 10 (worst pain)	Walker B.J. et al., 2019 [32]
PROMIS	Evaluates and monitors physical, mental, and social health in adults and children	PROMIS measures are standardized, allowing evaluation of many domains, including pain, fatigue, emotional distress, physical functioning, and social role participation.	Cella D. et al., 2010 [33]

Abbreviations. Ref: reference; MIDAS: migraine disability assessment; PedMIDAS: pediatric migraine disability assessment tool; PROMIS: patient-reported outcomes measurement information system.

**Table 3:** List of tools used to assess sleep disorders in the considered papers, with a brief description.

Tool	Description	Structure	Validated	Ref.
CSHQ	Parent-report sleep screening survey	33 three-point scale items grouped in 8 subscales relating to the major presenting clinical sleep complaints in pediatric age: bedtime resistance, sleep onset delay, sleep duration, sleep anxiety, night wakings, parasomnias, sleep-disordered breathing, and daytime sleepiness	Y	Markovich A.N. et al., 2015 [34]
BEARS	Pediatric sleep screening instrument	This includes 5 items: B = bedtime issues, E = excessive daytime sleepiness, A = night awakenings, R = regularity and duration of sleep, S = snoring	Y	Owens J.A. et al., 2005 [35]
SHIP	Instrument to target the sleep issues common in pediatric patients with headaches	This clearly differentiates between participants for whom sleep was and was not a clinical concern; positively correlates with anxiety, depression, and disability	Y	Rabner J. et al., 2017 [23]
SDSC	Parent-report sleep screening survey	26 items grouped in 6 subscales relating to the major presenting clinical sleep complaints in the pediatric age group: difficulty in initiating and maintaining sleep (DIMS), sleep breathing disorders (SBD), disorders of arousals/nightmares (DA), sleep/wake transition disorders (SWTD), disorders of excessive somnolence (DOES), and sleep hyperhidrosis (SHY). Each item is rated on a five-point scale	Y	Bruni O. et al., 1996 [36]
PDSS	Self-report questionnaire	Self-assessment instrument describing some daily life situations related to sleep habits, waking time, and sleep problems	Y	Badia P. et al., 2003 [37]

Abbreviations. CSHQ: children’s sleep habits questionnaire; BEARS: B = bedtime issues, E = excessive daytime sleepiness, A = night awakenings, R = regularity and duration of sleep, S = snoring; SHIP: sleep hygiene inventory for pediatrics; SDSC: sleep disturbance scale for children; PDSS: pediatric daytime sleepiness scale.

## References

[1] Abu-Arefeh I., Russell G. (1994). Prevalence of Headache and Migraine in Schoolchildren. BMJ.

[2] Owens J.A., Witmans M. (2004). Sleep Problems. Curr. Probl. Pediatr. Adolesc. Health Care.

[3] Rains J.C. (2018). Sleep and Migraine: Assessment and Treatment of Comorbid Sleep Disorders. Headache.

[4] Viticchi G., Falsetti L., Paolucci M., Altamura C., Buratti L., Salvemini S., Brunelli N., Bartolini M., Vernieri F., Silvestrini M. (2019). Influence of Chronotype on Migraine Characteristics. Neurol. Sci..

[5] Viticchi G., Altamura C., Falsetti L., Buratti L., Salvemini S., Polidoro F., Silvestrini M., Vernieri F., Bartolini M. (2020). Poor Sleep Quality in Patients Affected by Migraine: A Population Study. Neurol. Sci..

[6] Vgontzas A., Pavlović J.M. (2018). Sleep Disorders and Migraine: Review of Literature and Potential Pathophysiology Mechanisms. Headache.

[7] D’Andrea G., Cevoli S., Colavito D., Leon A. (2015). Biochemistry of Primary Headaches: Role of Tyrosine and Tryptophan Metabolism. Neurol. Sci..

[8] Esposito M., Parisi P., Miano S., Carotenuto M. (2013). Migraine and Periodic Limb Movement Disorders in Sleep in Children: A Preliminary Case-Control Study. J. Headache Pain.

[9] Miller V.A., Palermo T.M., Powers S.W., Scher M.S., Hershey A.D. (2003). Migraine Headaches and Sleep Disturbances in Children. Headache.

[10] Cheraghi F., Shamsaei F., Fayyazi A., Molaaei Yeganeh F., Roshanaei G. (2018). Comparison of the Quality of Sleep and Intensity of Headache between Migraine, Tension Headache, and Healthy Children. Iran. J. Child. Neurol..

[11] Guidetti V., Dosi C., Bruni O. (2014). The Relationship between Sleep and Headache in Children: Implications for Treatment. Cephalalgia.

[12] Nesbitt A.D., Leschziner G.D., Peatfield R.C. (2014). Headache, Drugs and Sleep. Cephalalgia.

[13] Lateef T., Witonsky K., He J., Ries Merikangas K. (2019). Headaches and Sleep Problems in US Adolescents: Findings from the National Comorbidity Survey—Adolescent Supplement (NCS-A). Cephalalgia.

[14] Nita S.A., Teleanu R.I., Bajenaru O.A. (2020). The Role of Polysomnography in Identifying Sleep Disorders in Children with Migraine. J. Med. Life.

[15] Roccella M., Marotta R., Operto F.F., Smirni D., Precenzano F., Bitetti I., Messina G., Sessa F., Di Mizio G., Loreto C. (2019). NREM Sleep Instability in Pediatric Migraine Without Aura. Front. Neurol..

[16] Vendrame M., Kaleyias J., Valencia I., Legido A., Kothare S.V. (2008). Polysomnographic Findings in Children with Headaches. Pediatr. Neurol..

[17] Moher D., Liberati A., Tetzlaff J., Altman D.G., PRISMA Group (2009). Preferred Reporting Items for Systematic Reviews and Meta-Analyses: The PRISMA Statement. PLoS Med..

[18] Methley A.M., Campbell S., Chew-Graham C., McNally R., Cheraghi-Sohi S. (2014). PICO, PICOS and SPIDER: A Comparison Study of Specificity and Sensitivity in Three Search Tools for Qualitative Systematic Reviews. BMC Health Serv. Res..

[19] Kemper K.J., Heyer G., Pakalnis A., Binkley P.F. (2016). What Factors Contribute to Headache-Related Disability in Teens?. Pediatr. Neurol..

[20] Torres-Ferrus M., Vila-Sala C., Quintana M., Ajanovic S., Gallardo V.J., Gomez J.B., Alvarez-Sabin J., Macaya A., Pozo-Rosich P. (2019). Headache, Comorbidities and Lifestyle in an Adolescent Population (The TEENs Study). Cephalalgia.

[21] Fonseca E., Torres-Ferrús M., Gallardo V.J., Macaya A., Pozo-Rosich P. (2020). Impact of Puberty in Pediatric Migraine: A Pilot Prospective Study. J. Clin. Neurol..

[22] Rabner J., Kaczynski K.J., Simons L.E., LeBel A. (2018). Pediatric Headache and Sleep Disturbance: A Comparison of Diagnostic Groups. Headache.

[23] Rabner J., Kaczynski K.J., Simons L.E., Lebel A.A. (2017). The Sleep Hygiene Inventory for Pediatrics: Development and Validation of a New Measure of Sleep in a Sample of Children and Adolescents With Chronic Headache. J. Child. Neurol..

[24] Maltese A., Salerno M., Tripi G., Romano P., Ricciardi A., Di Folco A., Di Filippo T., Parisi L. (2017). Internalizing problems are related to sleep patterns disordered in children affected by primary headache. Acta Med. Mediterr..

[25] Heyer G.L., Rose S.C., Merison K., Perkins S.Q., Lee J.E.M. (2014). Specific Headache Factors Predict Sleep Disturbances among Youth with Migraine. Pediatr. Neurol..

[26] Abou-Khadra M.K., Kishk N.A., Shaker O.G., Hassan A. (2014). Urinary 6-Sulphatoxymelatonin Levels and Sleep Disorders in Children with Migraine. J. Child. Neurol..

[27] Yilmaz M., Pıçakçıefe M., Ozge A., Palalı I. (2013). Migraine and tension-type headache in schoolchildren in western of turkey. Acta Med. Mediterr..

[28] Esposito M., Roccella M., Parisi L., Gallai B., Carotenuto M. (2013). Hypersomnia in Children Affected by Migraine without Aura: A Questionnaire-Based Case-Control Study. Neuropsychiatr. Dis. Treat..

[29] Carotenuto M., Esposito M., Precenzano F., Castaldo L., Roccella M. (2011). Cosleeping in Childhood Migraine. Minerva Pediatr..

[30] Stewart W.F., Lipton R.B., Dowson A.J., Sawyer J. (2001). Development and Testing of the Migraine Disability Assessment (MIDAS) Questionnaire to Assess Headache-Related Disability. Neurology.

[31] Hershey A.D., Powers S.W., Vockell A.L., LeCates S., Kabbouche M.A., Maynard M.K. (2001). PedMIDAS: Development of a Questionnaire to Assess Disability of Migraines in Children. Neurology.

[32] Walker B.J., Polaner D.M., Berde C.B. (2019). Acute Pain. A Practice of Anesthesia for Infants and Children (Sixth Edition).

[33] Cella D., Riley W., Stone A., Rothrock N., Reeve B., Yount S., Amtmann D., Bode R., Buysse D., Choi S. (2010). The Patient-Reported Outcomes Measurement Information System (PROMIS) Developed and Tested Its First Wave of Adult Self-Reported Health Outcome Item Banks: 2005-2008. J. Clin. Epidemiol..

[34] Markovich A.N., Gendron M.A., Corkum P.V. (2014). Validating the Children’s Sleep Habits Questionnaire Against Polysomnography and Actigraphy in School-Aged Children. Front. Psychiatry.

[35] Owens J.A., Dalzell V. (2005). Use of the “BEARS” Sleep Screening Tool in a Pediatric Residents’ Continuity Clinic: A Pilot Study. Sleep Med..

[36] Bruni O., Ottaviano S., Guidetti V., Romoli M., Innocenzi M., Cortesi F., Giannotti F. (1996). The Sleep Disturbance Scale for Children (SDSC). Construction and Validation of an Instrument to Evaluate Sleep Disturbances in Childhood and Adolescence. J. Sleep Res..

[37] Drake C., Nickel C., Burduvali E., Roth T., Jefferson C., Pietro B. (2003). The Pediatric Daytime Sleepiness Scale (PDSS): Sleep Habits and School Outcomes in Middle-School Children. Sleep.

[38] Voci A., Bruni O., Ferilli M.A.N., Papetti L., Tarantino S., Ursitti F., Sforza G., Vigevano F., Mazzone L., Valeriani M. (2021). Sleep Disorders in Pediatric Migraine: A Questionnaire-Based Study. J. Clin. Med..

[39] Gelfand A.A. (2013). Migraine and Childhood Periodic Syndromes in Children and Adolescents. Curr. Opin. Neurol..

[40] (2018). Headache Classification Committee of the International Headache Society (IHS) The International Classification of Headache Disorders, 3rd Edition. Cephalalgia.

[41] Abu-Arafeh I., Gelfand A.A. (2021). The Childhood Migraine Syndrome. Nat. Rev. Neurol..

[42] Tarantino S., Capuano A., Torriero R., Citti M., Vollono C., Gentile S., Vigevano F., Valeriani M. (2014). Migraine Equivalents as Part of Migraine Syndrome in Childhood. Pediatr. Neurol..

[43] Bruni O., Fabrizi P., Ottaviano S., Cortesi F., Giannotti F., Guidetti V. (1997). Prevalence of Sleep Disorders in Childhood and Adolescence with Headache: A Case-Control Study. Cephalalgia.

